# Analysis of Lung Microbiota in Bronchoalveolar Lavage, Protected Brush and Sputum Samples from Subjects with Mild-To-Moderate Cystic Fibrosis Lung Disease

**DOI:** 10.1371/journal.pone.0149998

**Published:** 2016-03-04

**Authors:** Deborah A. Hogan, Sven D. Willger, Emily L. Dolben, Thomas H. Hampton, Bruce A. Stanton, Hilary G. Morrison, Mitchell L. Sogin, Julianna Czum, Alix Ashare

**Affiliations:** 1 Microbiology and Immunology, Geisel School of Medicine at Dartmouth, Hanover, NH, United States of America; 2 Josephine Bay Paul Center for Comparative Molecular Biology and Evolution, Marine Biological Laboratory, Woods Hole, MA, United States of America; 3 Department of Radiology, Dartmouth-Hitchcock Medical Center, Lebanon, NH, United States of America; 4 Pulmonary and Critical Care Medicine, Dartmouth-Hitchcock Medical Center, Lebanon, NH, United States of America; Queens University Belfast, IRELAND

## Abstract

Individuals with cystic fibrosis (CF) often acquire chronic lung infections that lead to irreversible damage. We sought to examine regional variation in the microbial communities in the lungs of individuals with mild-to-moderate CF lung disease, to examine the relationship between the local microbiota and local damage, and to determine the relationships between microbiota in samples taken directly from the lung and the microbiota in spontaneously expectorated sputum. In this initial study, nine stable, adult CF patients with an FEV_1_>50% underwent regional sampling of different lobes of the right lung by bronchoalveolar lavage (BAL) and protected brush (PB) sampling of mucus plugs. Sputum samples were obtained from six of the nine subjects immediately prior to the procedure. Microbial community analysis was performed on DNA extracted from these samples and the extent of damage in each lobe was quantified from a recent CT scan. The extent of damage observed in regions of the right lung did not correlate with specific microbial genera, levels of community diversity or composition, or bacterial genome copies per ml of BAL fluid. In all subjects, BAL fluid from different regions of the lung contained similar microbial communities. In eight out of nine subjects, PB samples from different regions of the lung were also similar in microbial community composition, and were similar to microbial communities in BAL fluid from the same lobe. Microbial communities in PB samples were more diverse than those in BAL samples, suggesting enrichment of some taxa in mucus plugs. To our knowledge, this study is the first to examine the microbiota in different regions of the CF lung in clinically stable individuals with mild-to-moderate CF-related lung disease.

## Introduction

Chronic lung infections associated with cystic fibrosis (CF) cause progressive lung damage concomitant with decreased lung function. CF-related bronchiectasis often develops in localized regions of the lung [1–3], and these regions of bronchiectasis worsen over time [1]. In addition, damage is often more prominent in the upper lobes of the lung, pathology is often more severe in the right lung, and disease is primarily observed in the proximal airways rather than the alveoli [4,5]. It is not yet known if the regional variation in CF-related lung disease, particularly during the early stages of disease, is due to regional differences in microbial pathogens, variation in the host response to infection, or a combination of the two. An increased understanding of the regional variation has the potential to guide the discovery of interventions that will slow the progression of lung disease.

Several studies have analyzed the regional microbiota in CF. A comparison of bronchoalveolar lavage fluid (BAL) from the right middle lobe and the lingula of the left lung in children under age 6 found interlobar differences in the presence or levels of microbes by culture [6]. Another study in children with CF (median age of 8.5 years) found that in subjects with focal lung disease, culture analysis of BAL fluid from the lobe with the most damage identified all of the pathogens that were present in BAL fluid from all five lobes. However, when lung damage was more diffuse, interlobar heterogeneity in the cultured pathogens was observed in 11 of the 33 subjects [6]. A study of ten explanted CF lungs with severe damage found that in seven subjects, all five lobes had the same microbe with the highest relative abundance indicating that one bacterium (either *Pseudomonas aeruginosa*, *Burkholderia cepacia* complex, or *Achromobacter*) was strongly predominant in all regions of the lung [7]. The remaining three sample sets contained two different bacteria in the lobar samples, and there was regional variation in their relative abundance [7]. While the studies above provide information on the regional microbiota in children, and on the distribution of pathogens in the lungs of individuals with severe disease, little is known about the regional heterogeneity in CF adults with mild-to-moderate lung damage. This information is needed to determine how best to treat individuals in order to halt the progressive bronchiectasis that is often more rapid in particular lobes [8].

The analysis of sputum samples by culture-dependent methods, which is the most common way to clinically evaluate the lung microbiome in CF, and culture-independent methods has revealed many important aspects of CF lung disease. For example, certain species or genera have been correlated with better or worse health [9–11], and there is an indication of an inverse relationship between disease status and diversity of the microbial communities in the lung; increased diversity is associated with better disease status [12,13]. Because of the heavy reliance on the characterization of the microbiome of sputum to inform clinical practice, it is critical to understand how the composition of the lung microbiota compares to that in sputum. Sputum is collected after travel through the upper respiratory tract. In addition, sputum is enriched in mucus plugs, which may have communities that differ from those in the open airways. In Goddard et al. [7], it was found that the microbial communities in severely compromised lungs were less diverse than the communities in sputum from the same subject collected on the day of lung explantation and regional analysis [7]. However, it is not yet known how well the microbiota in sputum reflects the microbial communities in the lungs (BAL fluid and sputum plugs) in individuals with mild or moderate lung disease.

In this study, we aimed to address three questions. First, is there regional variation in microbiota in the lungs of adults with mild-to-moderate CF lung disease? Second, is there a relationship between the local microbiota and extent of lung damage? Third, is the microbiota in sputum, BAL fluid and protected brush (PB) samples of mucus plugs similar or different? We collected expectorated sputum, regional BAL fluid and PB samples by bronchoscopy of the right lung, and information on regional damage by CT scan from nine individuals with an FEV_1_>50%. Analysis of the microbial communities within these samples was obtained using DNA-based methods.

## Materials and Methods

### Human Subjects

Subjects with CF (n = 9) were enrolled if they had an FEV_1_ (forced expiratory volume in 1 second) > 50% predicted in an exam within 4 weeks of the procedure. All subjects were clinically stable and in their baseline state of health, and had not had an exacerbation within the preceding four weeks. All subjects were non-smokers. Enrolled subjects had a chest CT scan within 6 months of enrollment in order to assess damage in different regions of the lung. All female subjects underwent a pregnancy test and were excluded if positive. This study was approved by Committee for the Protection of Human Subjects (CPHS) of the Geisel School of Medicine at Dartmouth (CPHS #22781).

### Sample Collection

Following written informed consent, subjects were asked to produce a sputum sample and six of the nine subjects were able to do so. Subjects then underwent flexible bronchoscopy with a two-bronchoscope methodology that has been described in detail [14]. Briefly, after local anesthesia with viscous lidocaine to the posterior pharynx and intravenous sedation, a bronchoscope was inserted transorally and advanced to just above the vocal cords. In two subjects, this bronchoscope was removed and the channel was washed with normal saline (20 ml) to collect a scope wash, which analyzed for microbes. Subsequently, in these two patients, a second bronchoscope was passed through the vocal cords. The remaining seven subjects underwent only a single bronchoscope procedure with no scope wash. BAL fluid and PB samples were obtained from tertiary airways in the right upper, middle and/or lower lobes (RUL, RML and RLL, respectively). The right lung was sampled as most subjects had more CT evidence of lung disease on the right based on Brody Scores. In all but one subject, visible mucus was present at the tertiary airway orifice of at least two lobes and this mucus was obtained in the PB sample. If mucus was present in only two airways, then we limited our sampling to those airways and obtained only two PB samples. After obtaining PB samples, BAL was performed sequentially in the RUL, RML, and RLL with 20 ml of sterile saline followed by 10 ml of air. In all subjects BAL fluid return was between 12 ml and 15 ml from the RUL and RML and between 10 ml and 12 ml from the RLL. Following the procedure, subjects were monitored until they were stable for discharge. An aliquot of the BAL fluid from the RUL was sent to the DHMC clinical microbiology laboratory for routine culture analysis. Ten microliter aliquots were also spread plated on YPD (20 g peptone, 10 g yeast extract, 20 g dextrose, and 20 g agar in 1 L of water) to assess the levels of fungi in subjects 6 and 9.

### Brody Score Assignment

All enrolled subjects had a CT scan within 6 months of enrollment. CT scans were scored by a thoracic radiologist for severity of lung damage in all of the lobes. The thoracic radiologist who viewed the scan was blinded to all other patient data. The extent of damage was quantified using the modified Brody scoring method that assesses bronchiectasis, peribronchial wall thickness, mucus plugging, air trapping, and parenchymal infiltrates [15]. The original Brody Score was modified to include hyperinflation of the lung rather than air trapping, as expiratory images were not routinely obtained. This modification of the Brody score has been validated previously [16,17]. Brody score assignments were as follows:

- Bronchiectasis = (extent in central lung + extent in peripheral lung) x average size multiplier
- Mucus plugging = extent in central lung + extent in peripheral lung
- Peribronchial wall thickening = (extent in central lung + extent in peripheral lung) x severity
- Parenchymal score = extent of dense opacity + extent of ground glass opacity + extent of cysts or bullae
- Hyperinflation score = extent of hyperinflation x appearance of hyperinflation

### DNA Recovery from BAL, PB and Sputum Samples

For BAL and sputum samples used for nucleic acid extraction, an aliquot of the sample was either removed for DNA extraction (described below) or the sample was stored in the original sputum cup used for collection. For the PB samples, the brush was cut to a length of approximately 1–1.5 cm then placed in a 2 ml tube. Samples were immediately frozen and stored at -80°C. Within 30 days of freezing, the frozen samples were placed directly into a lyophilization chamber for freeze drying (Labconco FreeZone Benchtop Freeze Dry System) which generally took 12–24 h for samples to completely dry. For samples dried in the sputum cup, the sample was gently disrupted and transferred into a 2 ml tube prior to bead beating. Cells within the sample were lysed in a Biospec Mini-Beadbeater-16^™^ (Biospec Products, Bartlesville, OK, USA) with five one-minute rounds with two minutes on ice between rounds. The beads used were equal amounts of 0.1 mm, 0.5 mm and 1 mm beads (Biospec Products, Bartlesville, OK, USA). For the PB samples, the brush remained in the tube during bead beating. The DNA was isolated as described in Willger et al. [18]. Briefly, the bead beaten samples were resuspended in 300 μl of TE+DTT (TE amended with DTT at a final concentration of 0.08% added from a 2% stock solution) containing lysozyme (3 mg/ml), and incubated for 30 min at 37°C. Cell Lysis buffer (500 μl) (Qiagen Puregene Core Kit B, Qiagen, Valencia, CA, USA) was added, and the mixture was incubated for 15 min at 80°C. To remove RNA, RNase (1.5 μl) (Qiagen, Valencia, CA, USA) was added and the samples were incubated for 30 min at 37°C. Lysates were chilled on ice for 1 min, 200 μl of Protein Precipitation Solution (Qiagen Puregene Core Kit B, Qiagen, Valencia, CA, USA) was added, and the solutions were mixed vigorously for 20 sec. Cell debris was sedimented by centrifugation at 13,000 rpm for 3 min, and the supernatant was transferred to a new 1.5 ml tube prior to addition of 600 μl of 100% isopropanol. After mixing by inversion, the DNA was precipitated by centrifugation at 13,000 rpm for 20 min. The DNA pellet was washed with 300 μl of 70% ethanol and air dried before resuspension in 100–200 μl of DNA Hydration Solution (Qiagen Puregene Core Kit B, Qiagen, Valencia, CA, USA). The DNA concentrations were measured using a Nanodrop 2000 (Thermo Scientific, Wilmington, DE, USA).

### Analysis of the Bacterial 16S rRNA and Fungal ITS1 Sequences

The bacterial communities in the BAL, PB and sputum samples were characterized by Illumina MiSeq 16S rRNA v4v5 amplicon sequencing as previously described in detail [19,20]. Briefly, we amplified the bacterial v4v5 region from each sample in triplicate 33 μl reactions using fusion primers to barcode each sample in a multiplexing strategy. We pooled the triplicates and verified successful amplification on a Caliper LabChip GX (Perkin Elmer, Waltham, MA, USA). We cleaned amplicon products with Agencourt Ampure XP PCR Purification Beads (Beckman Coulter, Brea, CA, USA). Barcoded products were pooled in equimolar concentrations according to the target size and quantification from Caliper LabChip GX. All PCR reactions contained 1x HiFi Buffer, 2 mM MgSO_4_, 0.02 U/μl Platinum Taq polymerase (Invitrogen), 0.2 mM each dNTPs (ThermoFisher Scientific, Milwaukee, WI, USA), and up to 5 ng template. The final pool was size selected to remove primer dimers and contaminating human 18S products (Pippin Prep, SageScience, Beverly, MA, USA), quantitated (Kapa Biosystems, Woburn, MA, USA) and sequenced on a MiSeq 2x250 nt sequencing run. Quality filtering and chimera checks were performed as described [19]. For the analysis of fungi in samples 6 and 9, the ITS1 region was amplified using the same PCR settings as described above, but using the ITS1_F, ITS_R and ITS1_mblb_Bar primers published in Willger et al. [18]. The sequencing of the barcoded ITS1 products was performed with the identical settings as for the bacterial 16S rRNA.

The raw sequencing data for both the bacterial v4v5 and fungal ITS1 are deposited in the Sequence Read Archive (National Center for Biotechnology Information BioProject PRJNA288589, Study SRP060025; US National Library of Medicine, Bethesda, MD, USA).

### Quantification of Bacterial 16S rRNA Gene Copy Number

For quantification of total bacterial copies of the 16S rRNA gene in BAL fluid, 100 ng of the total DNA recovered from 1 ml of fluid was used as template. A region of the 16S rRNA locus was amplified by qPCR, using the universal bacterial primers “total bacteria_F” (5’-GTGSTGCAYGGYTGTCGTCA-3’) and “total bacteria_R” (5’-ACGTCRTCCMCACCTTCCTC-3’) which were added to the amplification reaction at a final concentration of 0.2 μM [21,22]. qPCR was conducted in 10 μl reaction volumes with the SsoFast EvaGreen Supermix (Bio-Rad Laboratories, Hercules, CA, USA) in a CFX96 Real-Time PCR Detection System combined with a C1000 thermal cycler (Bio-Rad Laboratories, Hercules, CA, USA). All PCRs were done in duplicate, and data were analyzed with the CFX96 System gene expression software. Standard curves containing a known numbers of genome equivalents were used to calculate total genome numbers using a standard curve prepared from *Pseudomonas aeruginosa* genomic DNA.

### Bioinformatics, Processing and Statistical Analyses

Taxonomy was assigned to amplicon sequences with GAST [23] against a curated SILVA database [24]. To compare operational taxonomic unit (OTU) clustering performance, the default UCLUST method was used [25] at a 97% similarity threshold with minimum cluster size of 2 using quantitative insights into microbial ecology (QIIME) (v1.5) [26]. Visualization and Analysis of Microbial Population Structures (VAMPs) tool (vamps.mbl.edu) was used to visualize the data. To identify the most abundant genera for presentation, an algorithm was used to calculate the smallest number of genera needed to represent 85% of all genera in all BAL and PB samples from bacteria- dominated subjects (Subjects 1–5 and 7, 8). This calculation resulted in 10 the genera *Stenotrophomonas*, *Staphylococcus*, *Pseudomonas*, *Streptococcus*, *Prevotella*, *Veillonella*, *Achromobacter*, *Herbaspirillum*, *Hydrogenophaga* and *Arenimonas*. *Hydrogenophaga* and *Arenimonas*, were the least abundant of these species and were omitted from the legend. The remaining eight genera were then used in all bar plots. The subsequent analysis of diversity in PB and BAL samples was performed utilizing the “diversity” function with the “Simpson diversity” setting in the “vegan” package (v 2.2–1) [27], the Bray-Curtis distances were calculated using the “distance” function with “bray-curtis” setting, and the PCA analysis was performed using the “pco” function with default “euclidean” distance setting in the “ecodist” package (v 1.2.9) [28] in RStudio (v 0.98.994) using R (v 3.1.1). For a deeper analysis of the PB samples, we identified the taxa which represented 99% of total reads (the remaining 1% of reads corresponded to ~400 taxa with each present in miniscule read numbers), then deleted the ten most abundant genera to allow for a focus on the minor members of each community. Heat maps were created in R using rank-based scoring as well as z-scored means of read counts for different bacterial genera across all subjects utilizing the “heatmap.2” function in the “gplots” package (v2.14.2) [29]. The program Prism 6 (GraphPad, San Diego, CA, USA) was used for all statistical tests.

## Results

The goal of this study was to determine: 1) if the lung microbiota varied in consistent ways across lobes of the right lung in individuals with mild-to-moderate CF lung disease, 2) if the extent of local lung damage correlated with specific microbial community properties, and 3) how the microbiota in sputum reflected the lung microbiota in BAL and PB samples from the lower airways. Nine clinically stable CF subjects, who had not experienced a period of disease exacerbation within the previous four weeks, underwent regional bronchoscopic sampling of the right lung. All subjects had mild-to-moderate lung disease with lung functions ranging from FEV_1_ 50–92% predicted (Table 1).

**Table 1:** Patient Characteristics. Gender, lung function, parenchymal lung damage, and clinical microbiology results in study subjects.

Subject			Brody Score			RUL BAL Clinical
ID	Gender	FEV1	RUL	RML	RLL	Microbiology
#1	M	90%	18.0	9.0	9.0	*Pa*, MSSA, MAC
#2	M	72%	24.5	17.3	18.5	*Pa*
#3	F	85%	22.0	18.3	12.5	*Achromobacter*
#4	F	56%	18.8	12.5	8.3	*Pa*
#5	M	92%	20.8	10.5	10.8	MSSA
#6	M	66%	27.0	18.0	16.0	*Candida lusitaniae*
#7	F	63%	17.8	15.5	11.3	*Stenotrophomonas*
#8	M	50%	21.3	11.0	7.0	N/A
#9	M	84%	33.0	21.0	20.8	*Candida lusitaniae*

The Brody score, a composite assessment of bronchiectasis, peribronchial thickening, parenchymal disease, air trapping and mucus plugging (detailed information in S1 Table), was used to assess the level of lung damage in the right upper, middle and lower lobes (RUL, RML and RLL, respectively) in a CT scan taken within six months of the bronchoscopy procedure.

FEV_1_, forced expiratory volume in 1 second (% predicted) measured within four weeks of the bronchoscopy procedure. Clinical Microbiology Report results from analysis of RUL BAL are shown.

PA; *Pseudomonas aeruginosa*;

MSSA, methicillin-sensitive *Staphylococcus aureus*;

MAC, *Mycobacterium avium* complex;

N/A, not analyzed.

**Fig 1:**
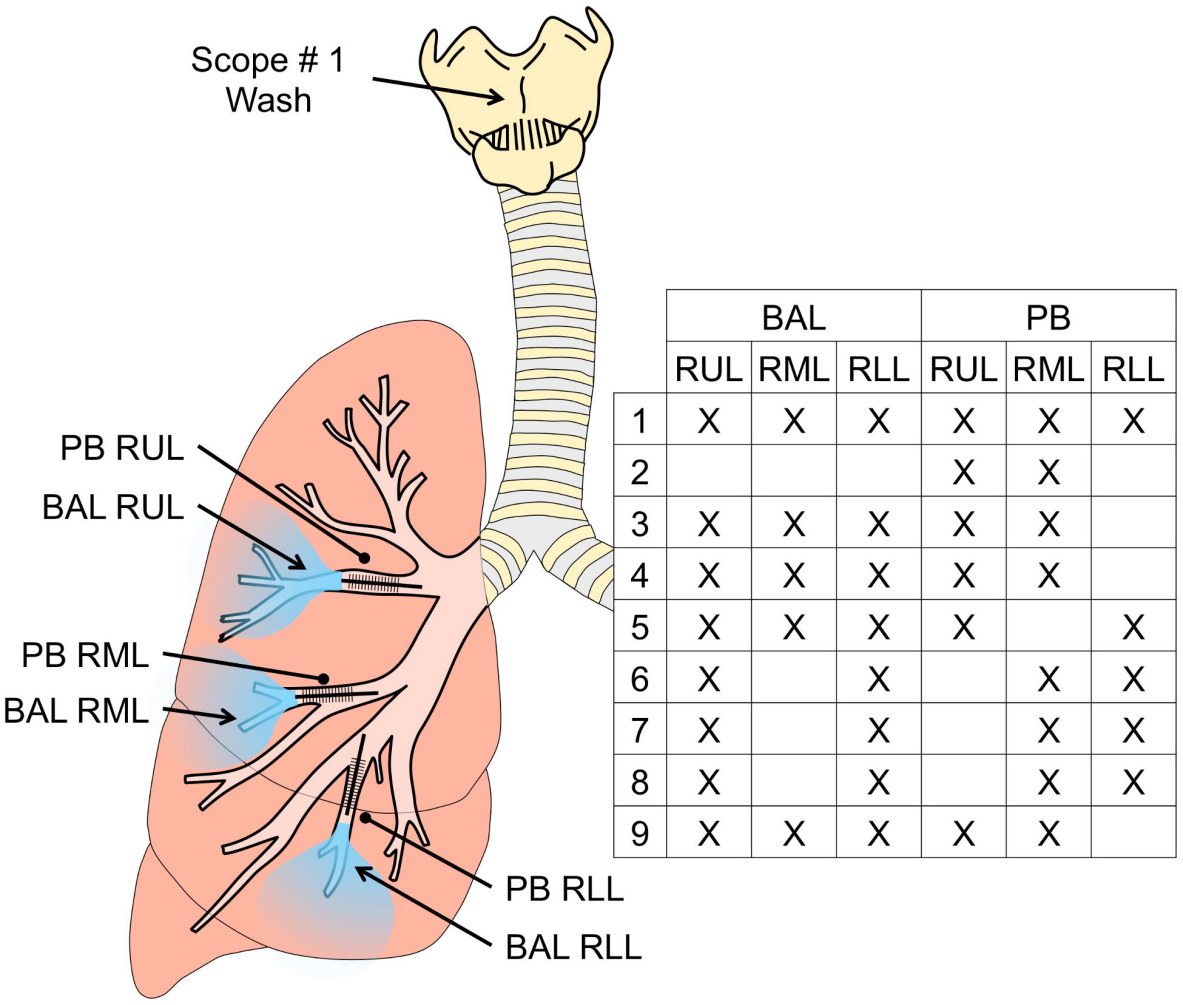
Regional sampling of the cystic fibrosis lung. PB samples were obtained from two or three tertiary bronchi of the right lung followed by sequential BAL sampling of the right upper lobe (RUL), right middle lobe (RML), and/or the right lower lobe (RLL). Specific sampling sites for each subject are shown within the Fig. For two subjects, a scope wash was also obtained prior to the lavage using a separate bronchoscope that was advanced to the glottis, removed, then washed through the suction channel to recover microbes for analysis.

All subjects had been colonized with the major pathogens seen in BAL culture for at least 6 months. For each individual, two PB samples of mucus were collected from the tertiary bronchi of two different lobes, followed by BAL of two or three lobes of the right lung (Fig 1).

If the subject was able to provide an expectorated sputum sample, this was collected prior to the bronchoscopic procedure. We also obtained data on the damage in the upper, middle and lower lobes in both lungs from each subject using a modified Brody score analysis of a recent computed tomography (CT) scan acquired less than 6 months prior to the bronchoscopy procedure [15–17,30] (Table 1). The pattern of damage as measured by Brody score analysis of the most recent CT scan (see Methods for details) was similar to previously published reports with the most extensive parenchymal lung damage in the RUL of the CF lung (P < 0.0001) [4,5,30,31], (Table 1 and S1 Table). This is discussed further below. The different components of the Brody score are discussed further below.

### Regional Uniformity of Microbiota in BAL Fluid and PB Samples within an Individual

**Fig 2:**
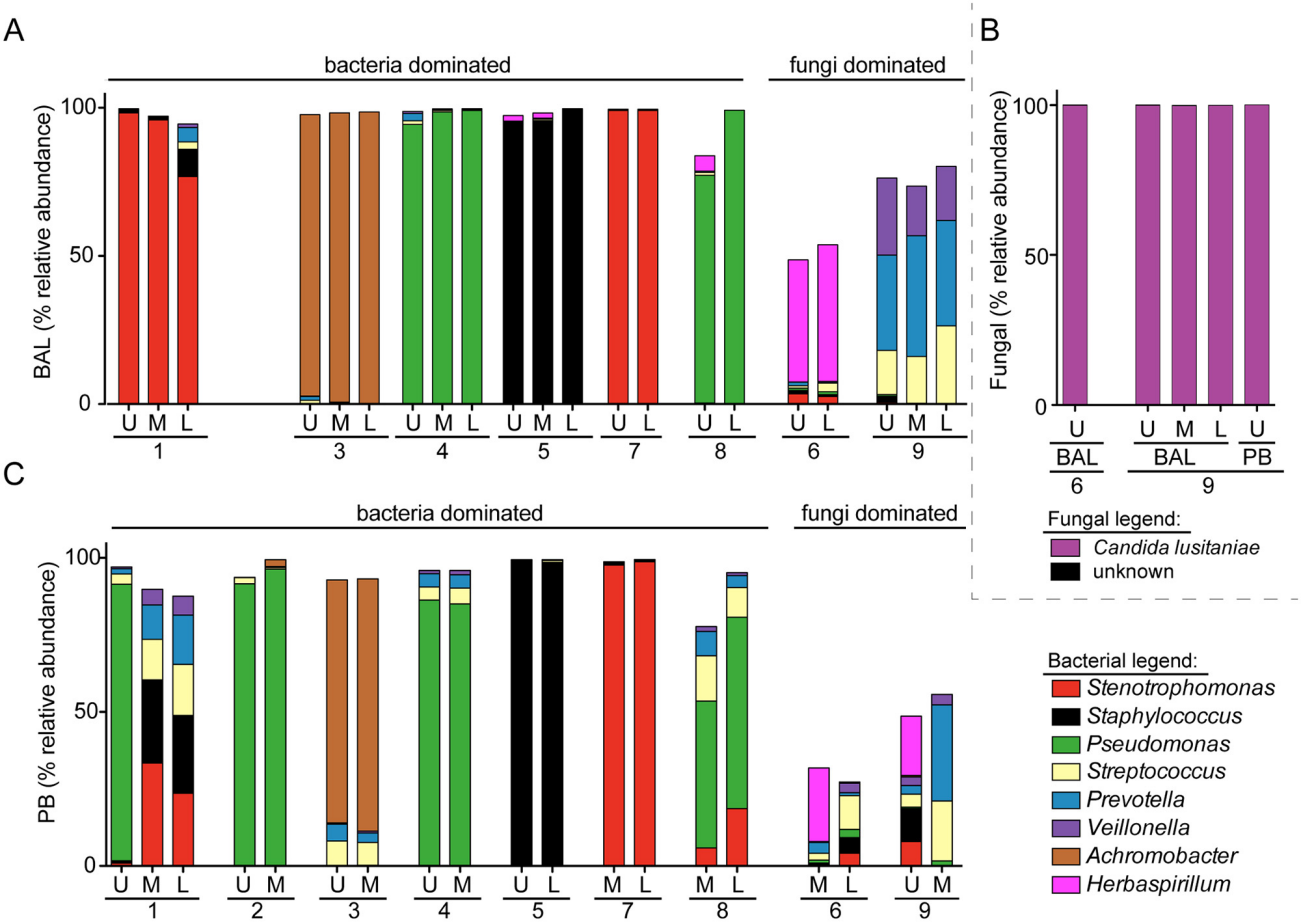
Analysis of the relative abundance of bacteria and fungi in BAL and PB samples from different lobes of the right lung. **A.** Relative abundance of bacterial genera in BAL samples from the upper (U), middle (M) and lower (L) lobes of the right lung in all subjects except for subject 2. Clinical microbiology reports found bacterial pathogens in the RUL BAL in subjects 1, 2, 3, 4, 5, 7 and 8 (listed in Table 1), and these are considered to be “bacteria dominated”. Clinical microbiology analysis of all subjects found predominantly *Candida lusitaniae* in the BAL fluid of subjects 6 and 9 and few if any bacteria, and these are therefore considered to be “fungi dominated”. The legend indicating the colors used to indicate the different genera in panels **A** and **C** is shown on the lower right. The taxa that made up 85% of all reads across all BAL and PB samples from “bacteria dominated” subjects are shown (data in S2 Table). **B**. Relative abundance of the fungal taxa in a subset of samples from subjects 6 and 9. *Candida lusitaniae* accounted for >99% of reads in these samples. **C**. Relative abundance of bacterial genera in PB samples from the upper (U), middle (M) and lower (L) lobes of the right lung.

To determine if there was regional heterogeneity in the microbiota in the BAL fluid from the lungs of individuals with CF with mild-to-moderate lung disease, microbial communities in lavage samples from two or three of the lobes of the right lung were compared. The bacterial microbiota in all samples was analyzed using Illumina-based sequencing of bacterial 16S rRNA gene amplicons; sequencing of amplified fungal ITS1 sequences was also performed for sample sets from two subjects. For seven of the nine patients analyzed (subjects 1, 3–5, 7, 8), the majority of BAL samples had one bacterial genus that made up more than 75% of total reads, and, within a subject, this dominant bacterial genus was the same in each lobe sampled (Fig 2A and S2 Table). Based on the high abundance of bacteria and the presence of a dominant bacterial genus we considered these samples “bacteria dominated” (Fig 2A). The most abundant genera detected in BAL of these seven subjects were common CF-related respiratory pathogens including *Pseudomonas* (2 subjects), *Stenotrophomonas* (2 subjects), *Achromobacter* (1 subject), and *Staphylococcus* (1 subject) (Fig 2A). The most abundant genera detected by 16S sequencing of DNA extracted from BAL matched the microbes found in the clinical microbiology analysis of RUL BAL fluid (Table 1) with one exception. In subject 1, RUL BAL cultured positive for *P*. *aeruginosa* and *Mycobacterium avium* complex, but had a very high relative abundance of *Stenotrophomonas* in the 16S rRNA sequence analysis of the RUL BAL. This discrepancy is discussed further below.

Two of the nine subjects (6 and 9) yielded BAL samples that by clinical culture on blood agar contained high levels of *Candida lusitaniae*, sometimes described under its teleomorph name *Clavispora lusitaniae* (Table 1), and few or no bacteria were detected in the clinical microbiology analysis (Table 1). Our laboratory cultures on YPD indicated that the *C*. *lusitaniae* abundance was between 1,000–10,000 colony forming units per ml of BAL from all sampled lobes from both subjects. These subjects were thus considered “fungi dominated”.

Analysis of the 16S rRNA gene sequences of the bacteria in BAL samples from subjects 6 and 9 found diverse bacterial genera without a predominant bacterial genus. Consistent with the microbiology analysis, none of the commonly encountered bacterial CF pathogens were abundant (Fig 2A). For samples from only the fungi dominated subjects, analysis of the mycobiome (fungal microbiota) was performed by amplification and sequencing of fungal ITS1 sequences. ITS1 sequence analysis found that *C*. *lusitaniae* was the most abundant genus making up >99% of all reads in both BAL and PB samples from both subjects (Fig 2B and S3 Table). *C*. *lusitaniae* is only rarely associated with CF lung disease [32,33], but is known as an opportunistic pathogen [34–36]. Together, these data suggest that a single bacterial (subjects 1, 2, 3, 4, 5, 7 and 8) or fungal (subjects 6 and 9) pathogen was the most abundant microbe in BAL samples from different lobes of the right lung in individuals with mild-to-moderate lung disease, and that the dominant microbe varied between subjects.

To complement the analysis of BAL samples from different regions of the lung, we performed a similar analysis of regional PB samples. In all subjects other than subject 1, the most abundant microbe in the PB samples (Fig 2C) was the same as in the BAL (Fig 2A).

**Fig 3:**
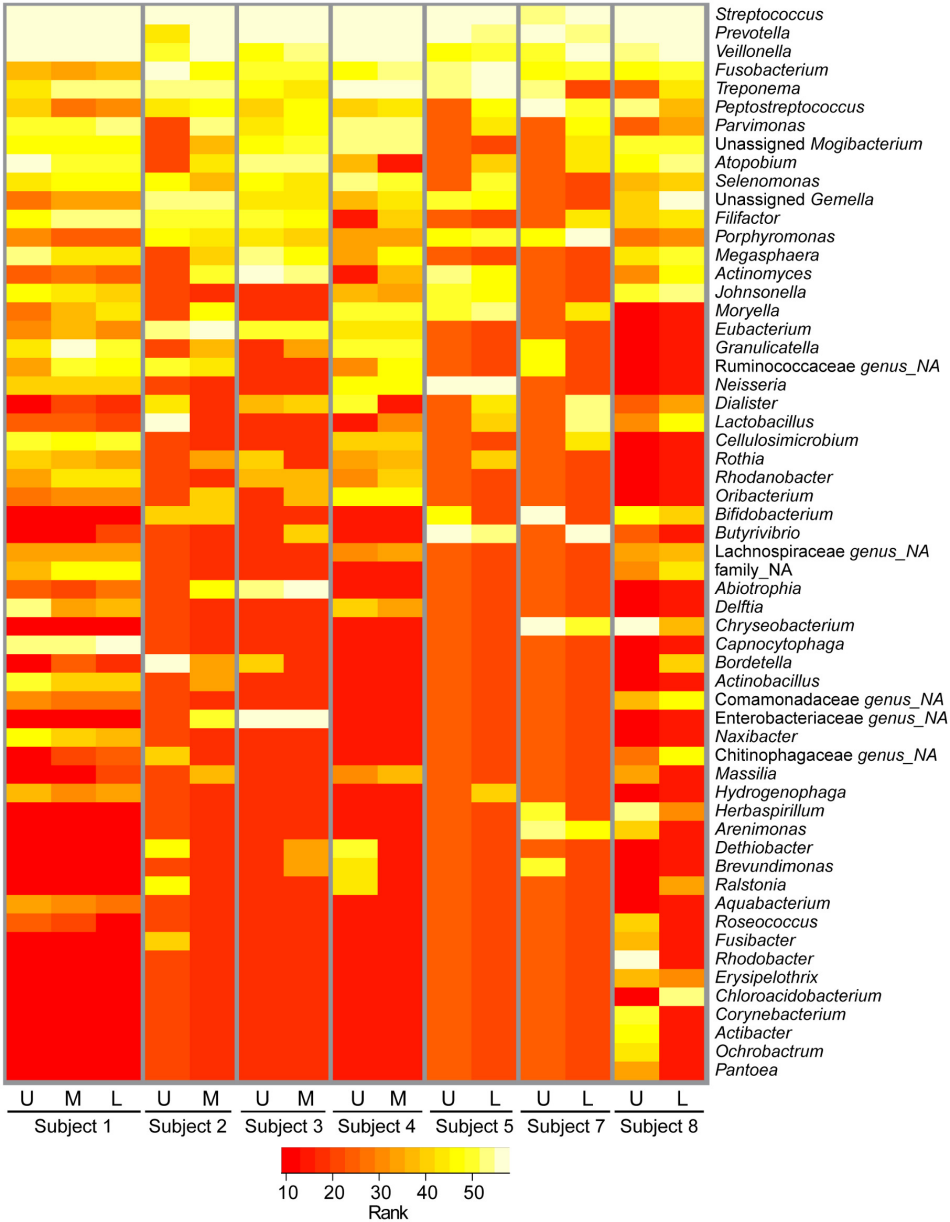
Rank abundance analysis of the less abundant taxa in PB samples. In this analysis, the known CF associated pathogens *Stenotrophomonas*, *Staphylococcus*, *Pseudomonas*, *Haemophilus* and *Achromobacter* were removed from the PB sample data to allow for a focus on the minor taxa (as described in the Methods). Rank abundance of the remaining taxa in those samples from the upper (U), middle (M) and lower (L) lobes of the right lungs of different subjects is shown. More abundant taxa are in yellow and less abundant taxa are in red as shown in the legend. Not all taxa are present in all samples and ranking all taxa across all samples resulted in the assignment of a rank within a sample even if a taxon is not present. This could lead to a visual effect, which suggest that some taxa are more abundant in a sample compared to others, even if they were undetected. For detailed information see S2 Table.

In subject 1, the RUL BAL and RUL PB had different predominant microbes in terms of relative abundance of amplified 16S rRNA gene sequences, with *Stenotrophomonas* dominating the BAL and *Pseudomonas* as the most abundant genus in the PB sample (Fig 2C). We found *Pseudomonas* at low relative abundance in the RUL BAL from subject 1 (0.01% of raw reads) and *Stenotrophomonas* at low relative abundance in the subject 1 RUL PB (0.9% of raw reads) indicating that both genera were present in both samples, but in strikingly different ratios. In addition, a member of the *M*. *avium* complex was detected in the RUL BAL by culture, while Illumina reads from the *Mycobacterium* genus were at low relative abundance in all samples from subject 1 (<0.01%, see Table 1 and S2 Table). In light of the concordance between the microbes with the highest relative abundance in all other matched BAL and PB samples obtained (compare Fig 2A and 2C), the difference between RUL BAL and PB samples from subject 1 was unexpected. To better understand the difference in relative abundances of *Stenotrophomonas* and *Pseudomonas* found in the RUL BAL and PB samples from Subject 1, we performed an analysis of the non-CF pathogens of the microbiota from the regional PBs by rank abundance (Fig 3). Despite the differences in the microbes with the highest relative abundance in the regional PBs from Subject 1 (Fig 2C), we found a consistent pattern for the non-pathogenic taxa in the PB samples from this subject (Fig 3). This provides strong confirmation of the fact that these samples did indeed originate from the same subject, and indicates that the differences were limited to most abundant species and not the minor members. While the rank abundance pattern for the minor taxa in terms of relative abundance was characteristic for subject 1, there was more variability among the minor taxa in the PBs from other subjects.

To better assess whether the minor members of the microflora reflect microbes or microbial DNA in the lung, or if its origin is oral contamination of the lung-derived BAL samples, we performed a “two bronchoscope” method on two subjects [37–40]. In this procedure, a bronchoscope that was passaged transorally to the glottis (Fig 1), immediately removed, and rinsed by suctioning sterile saline with the same volume of fluid used in a lavage procedure. This sample is referred to as a “scope wash” [14], and this sample represents the microbiome of the oral flora. A second bronchoscope was then used to collect PB and regional BAL samples. In this analysis of the taxa minus the known CF associated pathogens (*Stenotrophomonas*, *Staphylococcus*, *Pseudomonas*, *Haemophilus* and *Achromobacter*), we compared the scope wash microbiota to the microbial profiles in PBs and BALs, and found no visible similarity in patterns between the two (S1 Fig). Similarity between the BALs and the PBs also suggested that the BAL was not contaminated by oral flora as the protected brush samples were sheathed upon removal from the lung.

### Analysis of Microbiological Patterns across Lobes of the Right Lung within Subjects

**Fig 4:**
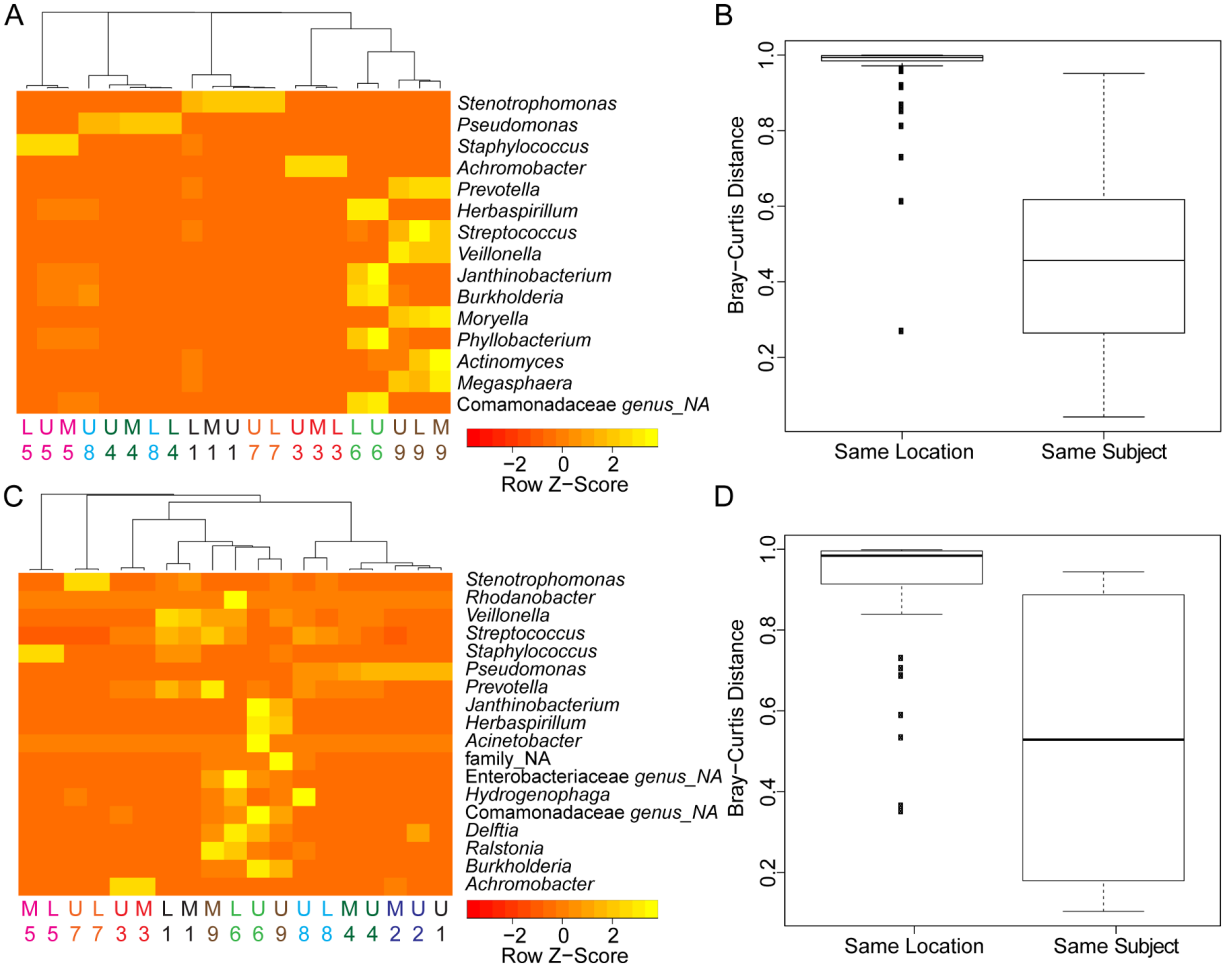
Analysis of relationships between the microbiota and location within the right lung. **A.** Cluster analysis of the relative abundance of BAL-associated taxa that accounted for more than 5% in any one sample. The numbers and colors indicate the subject. Genus was assigned in VAMPS except for taxa in the family Comamonadaceae for which a single genus could not be assigned (NA). **B.** Bray-Curtis distance analysis of BAL samples from the same location in different subjects (Same location), and samples from the different locations in the same subject (Same Subject) demonstrated that samples from the same subject are less different than samples from the same lobe in different subjects (*** P < 0.001, Wilcoxon Rank Sum Test). A Bray-Curtis Distance of 1 indicates essentially no relatedness, and a value of 0 indicates completely related. **C.** Cluster analysis of the relative abundance of PB-associated taxa that accounted for more than 5% in any one sample. The numbers and colors represent subject number. Taxonomic assignments were made using VAMPS. “NA” indicates that the indicated taxonomic level was not assigned. **D.** Bray-Curtis distance analysis found that PB samples from the *same location in different subjects* (Same Location) were more distant than samples from the *different locations in the same subject* (Same Subject) (*** P < 0.001, Wilcoxon Rank Sum Test). In **B** and **D**, the center line is the median, the box is interquartile range, and the whisker is 1.5 times the interquartile range from the median.

Focusing on taxa that represented more than 5% of the reads in any one BAL sample, we analyzed the microbiota by region in more detail. The relative abundances of taxa that comprised more than 5% of total reads were standardized by z-score for the purposes of data visualization. The z-score represents the relative abundance data in terms of the distance from the mean relative abundance for that taxon across all samples (Fig 4A). Samples were then clustered using Euclidian distance measurements. Samples clustered mainly by subject, and not by the region sampled, with the exception of clustering of the UL and LL samples from subjects 4 and 8, two subjects who were both colonized by *Pseudomonas* (Fig 4A). These data suggest that there was no evidence for enrichment for particular communities in different lobes of the lung across individuals. Visual analysis of the heat map also shows that no taxon was limited to one lobe of the lung across subjects. A Bray-Curtis distance analysis of the relatedness of BAL samples further confirmed that samples from the same subject were significantly more related to one another than samples from the same location in different subjects (Fig 4B, P < 0.001, Wilcoxon Rank Sum Test). Lastly, diversity, as measured by the Simpsons Diversity Index (SDI), was not significantly different from between lobes, though there was a slight, but not significant (P = 0.05554, Kruskal-Wallis test), trend towards increased diversity in the upper lobe relative to the middle and lower lobes (analysis not shown).

The PB samples were also used to determine if specific aspects of the microbiota were consistent across subjects in a specific region of the lung. Again, z-scored relative abundance data clustered by subject and not lobe (Fig 4C) and microbiome samples from different lobes within the subject were significantly more similar to each other than samples from the same location in different subjects in terms of Bray-Curtis distance (Fig 4D). Together, these data suggest that there is not a consistent pattern differentiating the different lobes, and this information is important to consider in light of the fact that CF lung disease progresses most rapidly in the RUL [5].

**Fig 5:**
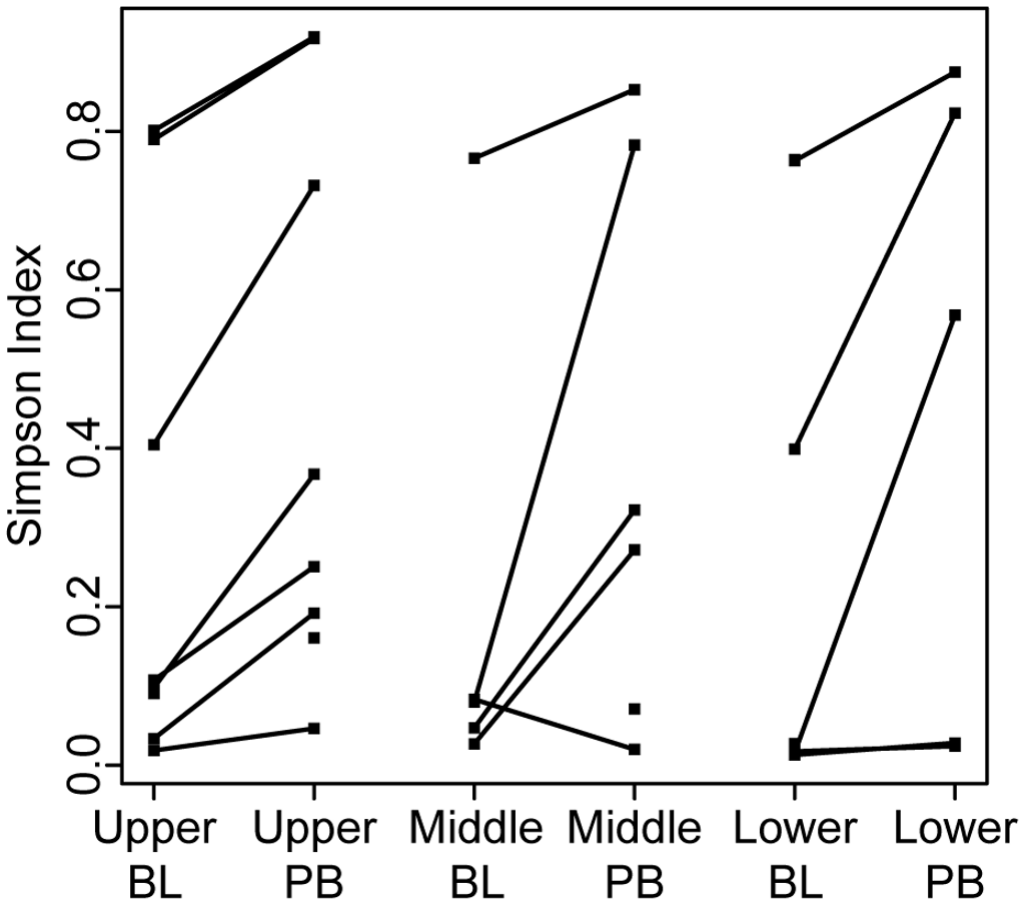
Bacterial Simpson Diversity Index for BAL- and PB-associated microbiota in the different lobes of the right lung. The diversity, as measured by Simpson Diversity Index, was calculated for each sample. When both a BAL (labeled BL) and PB sample was available from the lobe of a subject, their diversity values are connected with a line. The data used in this analysis are in S2 Table.

We further analyzed the relationship between the microbiota detected in BAL fluid, which samples both proximal and distal airways, and the microbiota detected in mucus plugs sampled by PB from a proximal airway segment in the same lobe (Fig 1). With the exception of the samples from the RUL from subject 1 (Fig 2A and 2C), PB and BAL samples had the same microbe in highest relative abundance. In addition, some of the less abundant microbes were consistently present at higher relative levels in PB samples than in BAL samples. Focusing on genera present in more than half of the samples, we found that *Streptococcus*, *Rothia*, *Peptostreptococcus*, and *Gemella* had higher relative abundances in the PB samples compared to the BAL samples when paired samples from the same lobe of the same subject were compared (P < 0.05, n = 17, paired t-test; analysis performed on data in S2 Table). In contrast, *Prevotella*, *Veillonella*, *Actinobacteria*, and *Fusobacteria*, were not significantly higher in relative abundance in PB samples when compared to BAL samples. A t-test of all pairs of samples for which there was both a BAL and a corresponding PB from the same lobe of the same subject shows that PB samples were more diverse, with a Simpson Diversity that is on average 0.23 greater in PB samples (Fig 5, P < 0.001). Together these data suggest that the microbiota within mucus plugs or in the proximal airways may be enriched in certain genera compared to the BAL. There were no genera that were only detected in one sample type.

### Relationship between the Sputum Microbiota and BAL and PB samples and Clinical Microbiology Data

**Fig 6:**
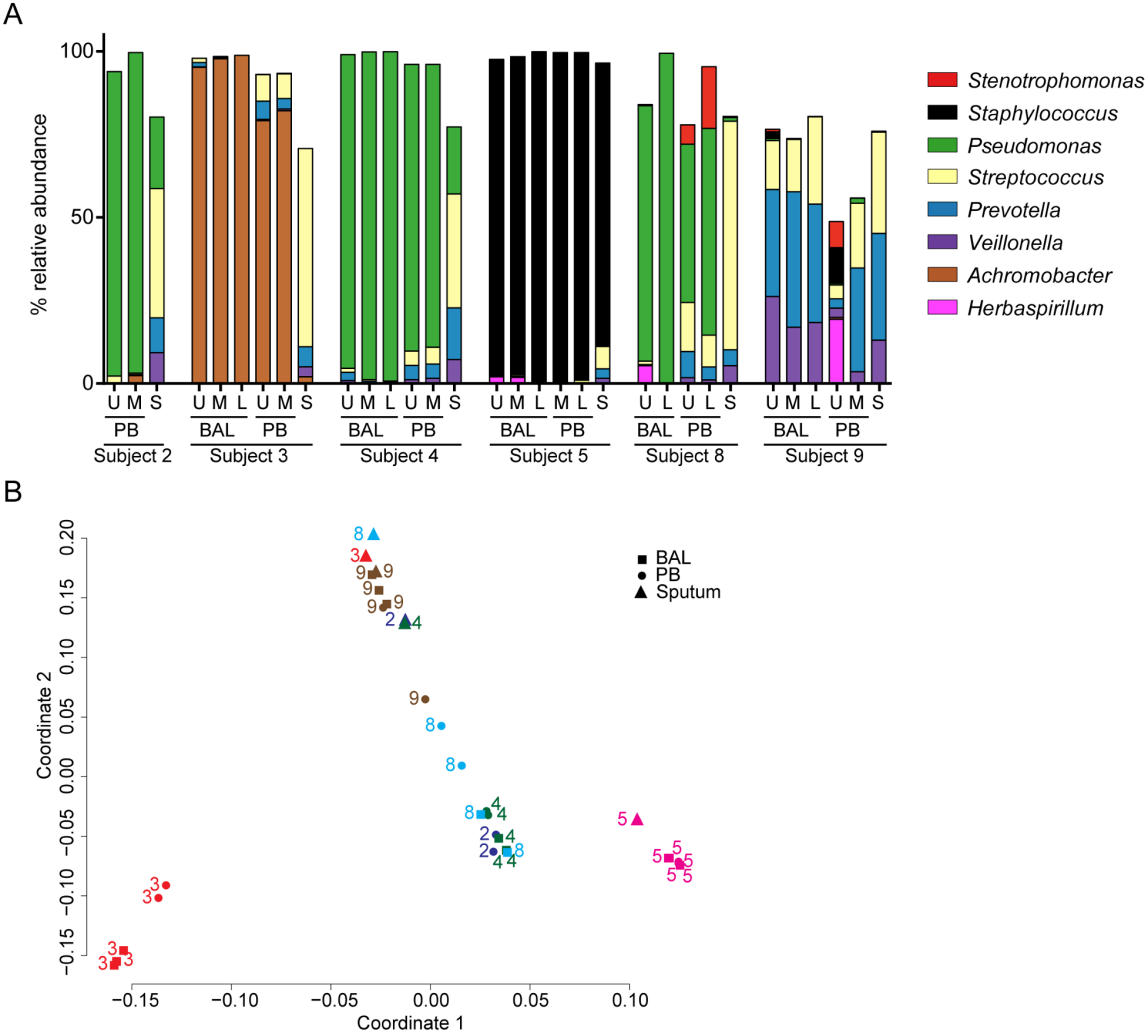
Comparison of sputum microbiota to regional PB and BAL fluid microbiota in samples from the same subject. **A.** Relative bacterial genus abundance in BAL and PB in the upper (U), middle (M) and lower (L) lobes of the right lung and in sputum. Data from the six subjects capable of providing a spontaneously expectorated sputum sample within 2 h of the bronchoscopy procedure are shown. **B.** Using the complete taxonomic data from the six subjects that provided a spontaneously expectorated sputum sample, a principal component analysis (PCA) was performed (see Methods for details). The numbers and colors indicate the subjects and the squares indicate BAL samples, the circles are from PB samples and the triangles show the sputum samples.

Sputum, which is believed to be comprised of lower airway-derived mucus plugs, is routinely expectorated, collected, and analyzed to gain insight into the microbes in the lung for the purposes of research and to guide clinical care. We determined how the microbial composition of sputum compared to that in BAL fluid and PB. Six subjects were capable of providing an expectorated sputum sample on the day of the bronchoscopic procedure for this analysis. In five out of six subjects, the sputum microbiota contained more genera at greater than 2% relative abundance than either the BAL or PB samples from the same subject (Friedman test, P < 0.01) (Fig 6A). These data suggest the community structure in sputum does not necessarily accurately represent the communities in the lung. The species that were notably higher in their relative abundance in sputum included *Streptococcus*, *Veillonella*, and *Prevotella*. *Streptococcus* was the only one of these species that was also significantly more abundant in PB samples of mucus plugs in comparison to BAL fluid from the same region.

For two of the six subjects who produced a sputum sample on the day of the bronchoscopic procedure, the microbe with the highest relative abundance in sputum was the same as that found in the BAL by both culture and DNA based methods (subject 5 with *S*. *aureus* and subject 9 with *C*. *lusitaniae*) (Fig 6A). For the other subjects (subjects 2, 3, 4 and 8), the organism with the highest relative abundance (>75% of total reads) in BAL samples was present in sputum, but was not the taxon at the highest relative abundance. When the sputum microbiota data were filtered to consider only the typical CF pathogens (*P*. *aeruginosa*, *Stenotrophomonas*, *Achromobacter*, *Burkholderia*, *Staphylococcus* and *Haemophilus*) (previously defined in [41–43]), the most abundant pathogen was the same in both sputum and lavage samples.

We used a principal component analysis (PCA) to compare sputum microbiota to those found by BAL and PB samples from the same subject (Fig 6B) and found that communities from sputum samples generally cluster together and are independent from the subject, whereas BAL and PB samples cluster together by subject. The exception are the samples from subject 5 that all cluster together, but that was not surprising since, as *S*. *aureus* was the most abundant organism in BAL, PB and sputum (Fig 6A). Interestingly, the samples of the fungi dominated subject 9 all cluster together with the sputum samples from all other subjects, indicating that in this subject there is no dominant bacterial genus and that highly diverse samples form their own cluster. Together, these data suggest that while sputum contains important information about lower airway infections, the community structure in sputum is not necessarily an accurate representation of the communities in the lung, due to the fact that sputum is a composite of mucus plugs from the lung and watery contribution from the oral flora when passing through the upper respiratory tract.

### Relationship between Parenchymal Lung Damage and Regional Microbiota

**Fig 7:**
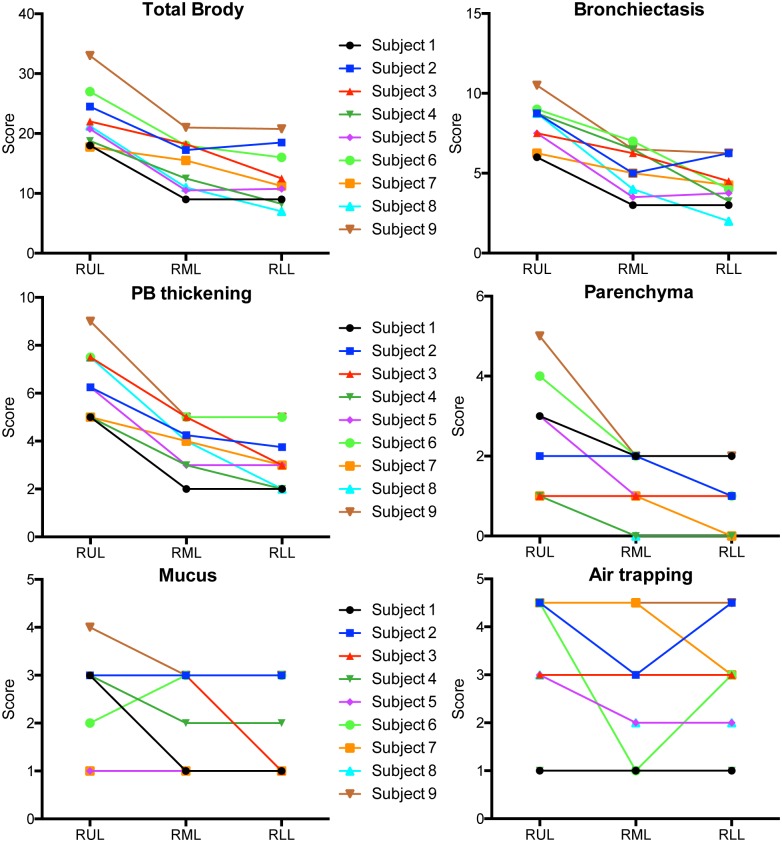
Scores of the single components for the Brody score to assess lung damage. The total Brody score is a composite of the evaluation of changes in bronchiectasis, peribronchial wall thickening (PB thickening), mucus plugging (Mucus), air trapping and parenchymal involvement (Parenchyma) in the lung of CF subjects.

The Brody score is a composite of the evaluation of the following morphologic changes: bronchiectasis, peribronchial wall thickening, mucus plugging, air trapping and parenchymal involvement [15]. When the components of the Brody score are analyzed separately they indicate that bronchiectasis and the peribronchial wall thickening (PB thickening) are the major drivers of the total Brody score (Fig 7). The most damage can be observed in the upper lobe (RUL) while the damage in the middle (RML) and lower lobe (RLL) is comparable. The parenchymal involvement (Parenchyma) score shows a similar trend but the impact on the total score is smaller. The mucus plugging and air trapping are more general lung phenomena and they don’t seem to be involved in the increased damage of the RUL in all CF subjects. For the values that comprise the composite Brody score, see the data in S1 Table. Similarly, the differences between some of the components of the Brody score over different regions of the lung (Fig 7) combined with the similarity in the microbiome in different regions of the lung (Fig 2) in the different lobes highlights the absence of any specific correlations between any of the components of the Brody score and community composition.

**Fig 8:**
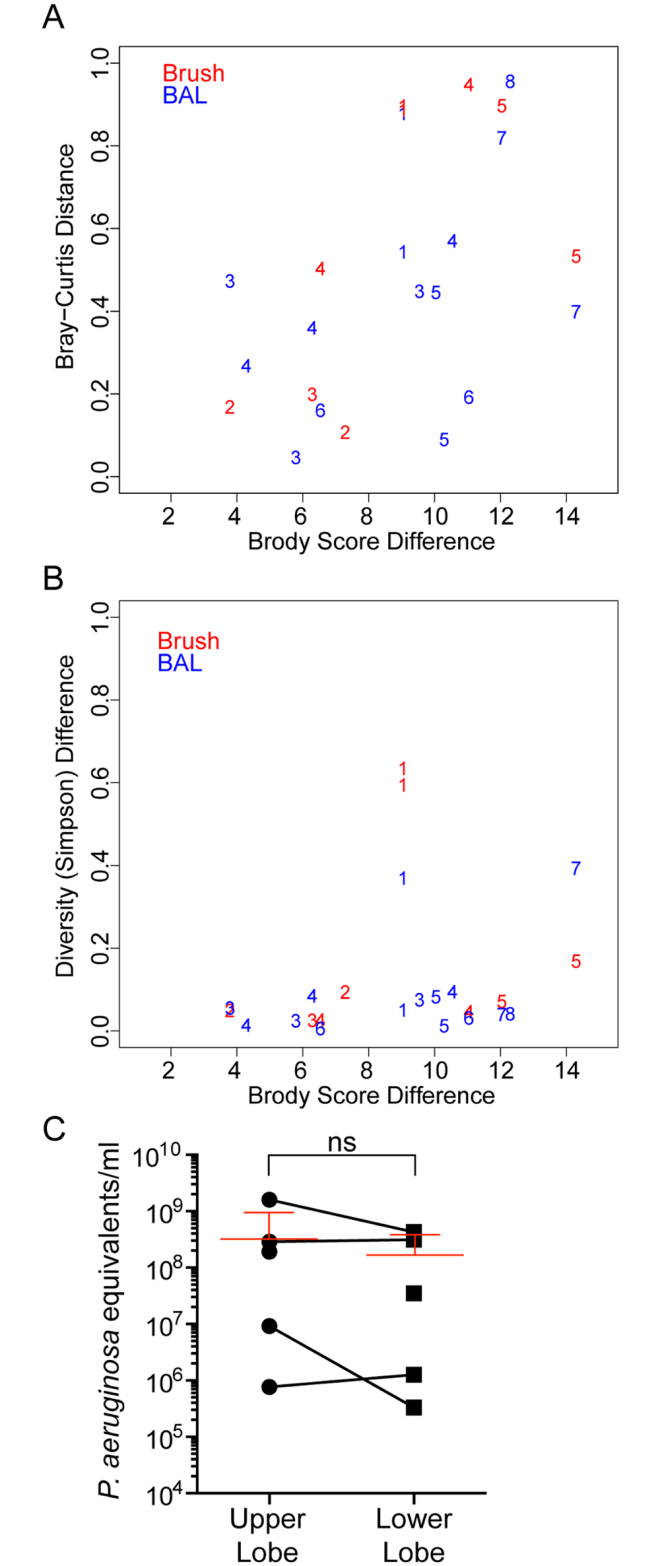
Relationship between lung damage and microbiota. **A.** Brody scores, a measure of lung damage, were calculated by a thoracic radiologist from a recent CT scan (data in S1 Table and Fig 7). The difference in Brody score between different lobes within a subject was calculated versus the difference in microbiota, determined as Bray-Curtis distance, is plotted for both BAL (red) or PB (blue) samples. The numbers indicate the data points from each of the subjects. **B.** A similar analysis as that shown in **A**, except that the Brody score difference between lobes within a subject is plotted against the difference in microbiota diversity, measured as the difference in Simpsons Diversity Index, in BAL or PB samples in the lobes being compared. **C.** Quantification of the amount of bacterial DNA in BAL fluid from the right upper lobe and right lower lobe. The red lines indicate the mean of all samples including error bars.

We were interested if the increased damage in the RUL can be attributed to any microbial contribution be it the presence of a certain bacterial genus or the degree of diversity that was observed. When the microbiota in the BAL and PB samples were analyzed in comparison to the extent of damage (Brody Score), there was no correlation between Brody score and sample composition as analyzed by Bray-Curtis distance across all samples (Fig 8A). This result was not surprising in light of the large differences in community composition and predominant taxa across subjects (Fig 3). To control for intersubject variability, we analyzed the difference in the Brody score between lobes from the same subject and the differences in the microbiota within the BAL from those same regions. This analysis found a significant correlation between interlobar difference in Brody score and Bray-Curtis distance (P ≤ 0.05) suggesting that extent of lung damage within a subject may influence the bacterial community composition to a modest extent or that different community compositions differentially impact lung damage (Fig 8A). We did not find a correlation between differences in Brody score and differences in microbiota diversity measured by Simpson diversity (Fig 8B).

Focusing on the differences between the RUL and the RLL, which show the greatest different in Brody score, we sought to determine if there was a difference in total bacterial load in BAL fluid recovered from these lobes. Quantitation of the bacterial burden in the right upper and lower lobes by *P*. *aeruginosa* 16S rRNA gene copies per ml of BAL fluid using quantitative PCR did not find a pattern in bacterial loads in the RUL in comparison to the RLL (Fig 8C). We are aware that our quantification approach is not taking into account that there are differences in the amount of 16S copies per organism [44] and that the metabolic state of the cells is affecting the copy number [45], but since the dominant genera per patient in the BAL fluid recovered from the upper and lower lung are the same (Fig 3A) and the metabolic state varies within a log_10_ we feel confident to conclude that there is no statistical significant change between the bacterial loads in the RUL in comparison to the RLL. Together, these data suggest that aspects of the host-microbe interaction, and not differences in the microbial communities themselves, drive the more rapid progression of CF lung disease in the RUL, as discussed next.

## Discussion

These studies represent the first analysis of the regional microbiota in the lungs of adult CF patients with mild-to-moderate lung disease during periods of clinical stability. Our data found that the extent of damage in regions of the right lungs of subjects with CF did not correlate with specific microbial genera, levels of community diversity or composition, or bacterial genome copies per ml of BAL fluid. These studies also found that in all cases, BALs from different regions contained similar microbial communities, based on a variety of measures. In eight out of nine subjects, PB samplings from different regions of the same subject’s lung were also similar in microbial community composition and similar to communities in BAL fluid from the same lobe. Microbial communities in PB samples were more diverse than those in BAL samples, with more species with >5% relative abundance, suggesting the enrichment of certain taxa in mucus plugs.

Cystic fibrosis transmembrane conductance regulator (CFTR) dysfunction in airway epithelia and other cells has multiple clinical implications including dehydration of the airway surface liquid [46], hyperinflammation [47], abnormal airway pH [48], impaired immune cell function [49], impaired activity of antimicrobial factors [50], and chronic infection. The combination of these effects favors the development of mucus plugging within the airways that is both transient and diffuse within the proximal airways of the CF lung [51,52]. The microbiota in the PB samples of mucus plugs differed from BAL samples from the same region in that they were more diverse using the Simpson Diversity Index metric and there were more taxa with greater than 5% percent relative abundance in any one sample. These findings are consistent with those from studies of ventilator-associated pneumonia (VAP), in which there were higher levels of oral flora in more proximal samples, as detected by standard culture [53,54]. *Streptococcus* spp. were among the microbial genera with significantly higher relative abundance in PB samples when compared to BAL. *Rothia*, which was also significantly enriched in PB samples has been reported to be a common CF opportunist [55]. While higher relative abundances of *Prevotella* in PB versus BAL samples was not significant across all sample pairs, several subjects had notably higher levels of *Prevotella* in PB samples in comparison to BAL from the same lobe (Fig 3). The presence of *Prevotella* in the lung is no indication that this genus is involved causing disease in CF since *Prevotella* has been reported in both CF and non-CF samples from the lower airways, and higher levels of this genus were seen in samples from individuals with CF [56,57]. Rogers and colleagues also found that anaerobes such as *Prevotella* were present in the non-CF bronchiectatic lung [58].

In samples from the RUL of one subject, *P*. *aeruginosa* was found to be abundant in the PB sample but *Stenotrophomonas* was the highest abundance microbe in the BAL fluid (Fig 2). This observation was similar to a pattern detected in the analysis of an explanted lung affected with COPD in which *P*. *aeruginosa* was found in the upper middle bronchus, but *Stenotrophomonas* was found in the distal bronchus from the same lung [59]. In both our study and the analysis of regional microbiota in COPD [59], variation in bacterial community composition among regional samples was only seen in one subject; other subjects had more uniform microbial communities throughout the lung. Interestingly, in a study that analyzed the microbes in BAL fluid from all six lobes in children with CF, *Stenotrophomonas* stood out as the microbe most likely to be localized to only one or two lobes of the lung [6].

This study provides further support for the use of sputum as a useful sample for the diagnosis of the most abundant pathogens in CF lung infections in most subjects. However, our results suggest that sputum diversity may not always reflect the diversity of the lung microbiome in the lower airways. In addition, the sputum microbiota contained higher levels of some taxa that were more abundant in PB samples relative to BAL samples. For example, *Streptococcus* and *Prevotella* in subjects 3, 4, and 8 have a higher relative abundance in PB samples when compared to BAL samples and yet higher relative abundances in sputum (Fig 6A). Our studies also parallel a study reports by Singh and colleagues [7], which showed that in patients with end stage disease, the microbial diversity in samples from the lung was lower than in sputum. While our data demonstrate that the most abundant pathogen in sputum generally reflects the predominant taxa identified in samples from the right lung, a limitation of our study was that only one lung was sampled. However, in our study the relative uniformity between the different lobes from the same lung lends support for the use of sputum, which may come from any lobe or multiple lobes, in clinical diagnosis. Furthermore, it provides important insight into how different methods of sampling the lung (BAL vs. PB) may impact study interpretation and clinical diagnoses. Bronchoscopy did not yield any new genera beyond those identified in sputum.

The presence of high levels of *C*. *lusitaniae* in the CF lung was surprising. While numerous studies have documented the presence of *Candida* spp. in the lung, the most commonly detected species are *Candida albicans*, *Candida parapsilosis*, and *Candida dubliniensis* [18,33,60,61]. The lavage samples containing *C*. *lusitaniae* had high fungal loads (>10^5^ CFUs per ml of BAL fluid) suggesting that this species has the potential to thrive in the CF lung. In the two fungus-dominated subjects, the levels of bacteria as measured by plating and by quantitativate PCR of the 16S rRNA gene was lower than for the bacterially dominated subjects, and no single bacterial genus predominated. *C*. *lusitaniae* has been detected in CF samples previously, but is uncommon and/or usually at low levels [18,32,33,62]. As was seen in the regional samples from subjects with predominantly bacterial infections, *C*. *lusitaniae* was found in all lobes sampled.

While Dickson et al. [40] found higher bacterial diversity in the upper lobes compared to the lower lobes in healthy individuals, we did not observe statistically significant differences in diversity in different lobes of the right lung. The absence of a significant link between regional lung damage and any of the microbiological parameters measured here suggests that it is the host-microbe interaction, rather than the nature of the microbial infection itself, that promotes lung damage. Recent studies on isolates recovered from explanted lungs with late stage disease suggest that there are geographic differences in microbial genotype and phenotype in the different regions of the lung and this may contribute to the regional variation in damage [7,63,64]. Future studies will determine if microbial phenotype also varies regionally in individuals with less advanced disease.

To our knowledge, this study is the first to examine the microbiota in different regions of the CF lung in clinically stable individuals with mild-to-moderate CF-related lung disease. We report that the extent of damage observed in regions of the right lung did not correlate with specific microbial parameters and that the microbiota across the right lung was similar when BAL samples were compared, and only one subject had regional heterogeneity in the microbiota of the right lung when PB samples were analyzed. Microbial communities in PB samples were more diverse than those in BAL samples, suggesting enrichment of some taxa in mucus plugs. Future studies will focus on determining how the microbiota contributes to the heterogeneous progression of lung disease in CF.

## Supporting Information

S1 Fig Rank abundance analysis of the less abundant taxa in BAL, PB and scope wash samples. In this analysis, the known CF associated pathogens *Stenotrophomonas*, *Staphylococcus*, *Pseudomonas*, *Haemophilus* and *Achromobacter* were removed from the BAL, PB and “scope wash” sample data to allow for a focus on the minor taxa (as described in the Methods). Rank abundance of the remaining taxa in those samples from the upper (U), middle (M) and lower (L) lobes of the right lungs and “scope wash” (W) of different subjects is shown. More abundant taxa are in yellow and less abundant taxa are in red as shown in the legend
(TIF)

S1 Table Components of the Brody score. Presented are the total Brody scores of the three right lobes of the study subjects, including the scores for each component of the Brody score that evaluate changes in bronchiectasis, peribronchial wall thickening (PB thickening), mucus plugging (Mucus), air trapping and parenchymal involvement (Parenchyma) in the lung of CF subjects.
(XLSX)

S2 Table Illumina analysis of the bacterial 16S rRNA sequences amplified from total DNA isolated from lung-derived and scope wash samples. The numbers represent the absolute number of reads (left) and the relative abundance in percent (right) for each sample. Reads were given a taxonomic assignment using VAMPS (www.vamps.mbl.edu). The database selected was limited to the bacteria, and the raw numbers represent the absolute number of reads. The most abundant genera are color coded based on their representative colors in the bar plots.
(XLSX)

S3 Table Illumina analysis of the fungal ITS1 sequences amplified from total DNA isolated from a subset of lung-derived samples. The numbers represent the absolute number of reads on the left and the relative abundance in percent on the right from each sample. Reads were given a taxonomic assignment using VAMPS (www.vamps.mbl.edu). The database selected was limited to the Eukarya, and the numbers represent the absolute number of reads. The most abundant species are color coded as the most abundant species in Fig 3B.
(XLSX)
