# Supplementary figures and images for: Analysis of Lung Microbiota in Bronchoalveolar Lavage, Protected Brush and Sputum Samples from Subjects with Mild-To-Moderate Cystic Fibrosis Lung Disease
Source: PLoS One. 2016 Mar 4;11(3):e0149998. doi: 10.1371/journal.pone.0149998 (PMC4778801; doi:10.1371/journal.pone.0149998)

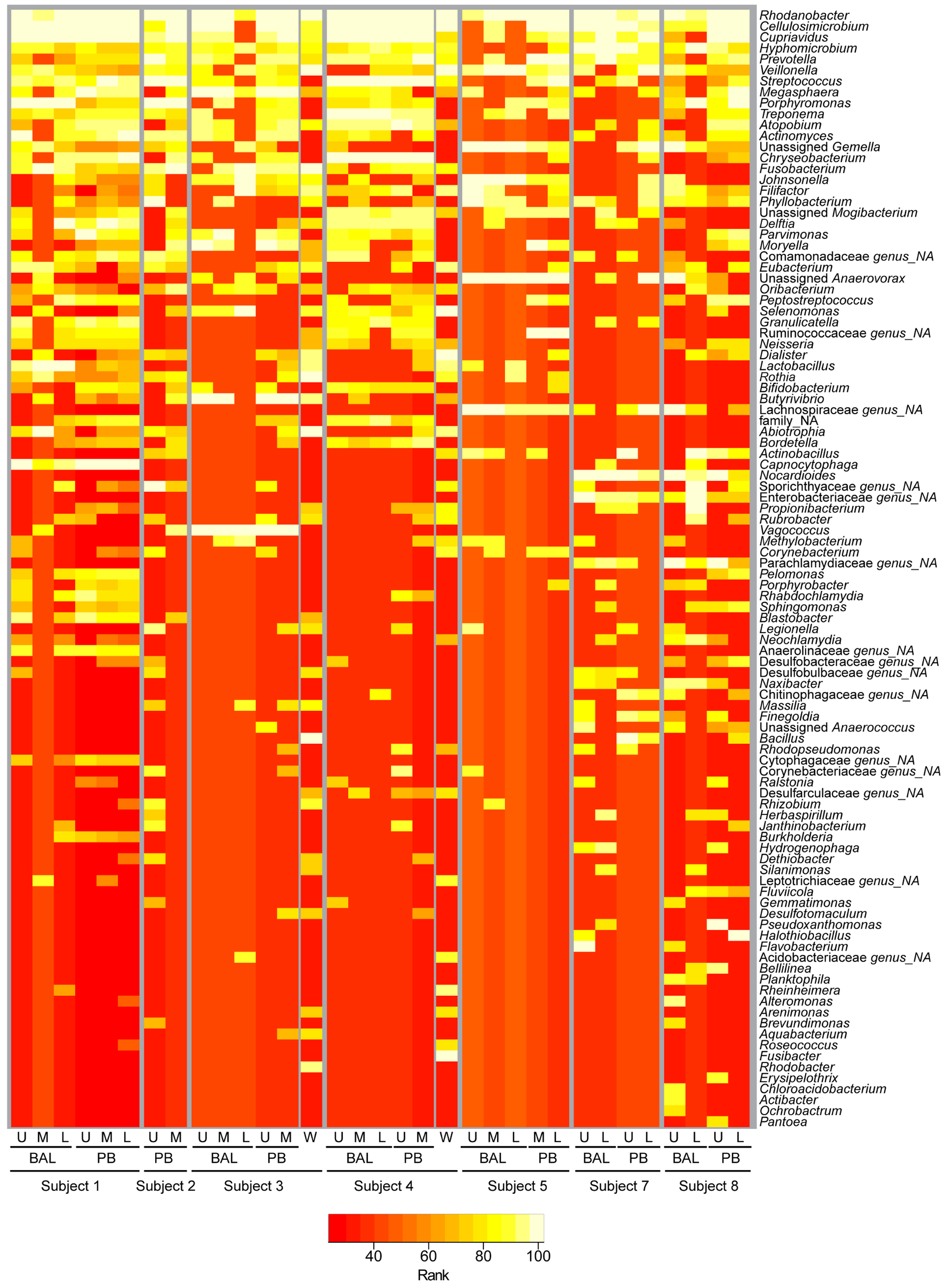

Supplement: S1 Fig — In this analysis, the known CF associated pathogens Stenotrophomonas, Staphylococcus, Pseudomonas, Haemophilus and Achromobacter were removed from the BAL, PB and “scope wash” sample data to allow for a focus on the minor taxa (as described in the Methods). Rank abundance of the remaining taxa in those samples from the upper (U), middle (M) and lower (L) lobes of the right lungs and “scope wash” (W) of different subjects is shown. More abundant taxa are in yellow and less abundant taxa are in red as shown in the legend (TIF) [file pone.0149998.s001.tif]
